# The perceived stability of scenes: serial dependence in ensemble representations

**DOI:** 10.1038/s41598-017-02201-5

**Published:** 2017-05-16

**Authors:** Mauro Manassi, Alina Liberman, Wesley Chaney, David Whitney

**Affiliations:** 1 Department of Psychology, University of California, Berkeley, CA USA; 2 Vision Science Group, University of California, Berkeley, CA USA; 3 Helen Wills Neuroscience Institute, University of California, Berkeley, CA USA

## Abstract

We are continuously surrounded by a noisy and ever-changing environment. Instead of analyzing all the elements in a scene, our visual system has the ability to compress an enormous amount of visual information into ensemble representations, such as perceiving a forest instead of every single tree. Still, it is unclear why such complex scenes appear to be the same from moment to moment despite fluctuations, noise, and discontinuities in retinal images. The general effects of change blindness are usually thought to stabilize scene perception, making us unaware of minor inconsistencies between scenes. Here, we propose an alternative, that stable scene perception is actively achieved by the visual system through global serial dependencies: the appearance of scene gist is sequentially dependent on the gist perceived in previous moments. To test this hypothesis, we used summary statistical information as a proxy for “gist” level, global information in a scene. We found evidence for serial dependence in summary statistical representations. Furthermore, we show that this kind of serial dependence occurs at the ensemble level, where local elements are already merged into global representations. Taken together, our results provide a mechanism through which serial dependence can promote the apparent consistency of scenes over time.

## Introduction

In everyday life, we are constantly surrounded by complex and cluttered scenes: the kitchen we live in, the garden we visit, the crowded street we walk on. Within a single scene, our visual system has to continuously switch from local representations, such as perceiving a single person, to global representations, such as perceiving a crowd of people. In fact, our visual system has the ability and natural tendency to represent sets of similar items using summary statistics^1–3^. While this ensemble coding heuristic has the disadvantage of losing precise information about each single item^4, 5^, it has the advantage of condensing extremely cluttered environments into global representations (e.g. perceiving a forest instead of each individual tree)^6, 7^.

Over time, the physical input from these complex scenes constantly change because our eyes, head and body move, and discountinues are introduced by eye blinks. Moreover, the scenes themselves are often dynamic and there are additional changes induced by lighting and noise fluctuations. It is therefore crucial for our visual system to stabilize representations of these complex scenes over time in order to achieve perceptual constancy of the environment in which we live. How this stabilization occurs is still largely unknown.

The general effects of change blindness and/or inattentional blindness might provide one means of stabilizing the appearance of scenes despite fluctuations in retinal images, noise, and discontinuities. In many change blindness experiments, participants are shown a scene which is then followed by another duplicate scene usually with a single change made to it (such as a new object, an object with a changed identity, etc.)^8–11^. Participants are asked to compare the two scenes and report the change. Surprisingly, participants often fail to immediately notice such changes. Inattentional blindness, generally speaking, is a failure to detect or recognize an unattended object or feature^10, 12, 13^. Simply in virtue of not noticing any change, change blindness and inattentional blindness may promote perceptual stability, i.e., the consistent appearance of similar scenes when viewed sequentially.

While inattention might contribute to the appearance of stability, it is an incomplete explanation for a few reasons. First, inattentional and change blindness generally refer to particular objects, and do not clearly involve the ensemble representation of a scene. Indeed, observers can suffer from change blindness for individual objects and yet perceive a change at the level of ensemble or summary statistical information in scenes^14^. Second, demonstrations of change and inattentional blindness show failures to notice changes in a scene, but they do not actually show that apparent stability is achieved between scenes. In other words, simply being blind to changes does not mean that scenes will appear to be the same.

Recently, a novel mechanism of object stabilization was proposed, suggesting that perception occurs through continuity fields: spatiotemporally tuned operators within which similar features and objects are integrated. Continuity fields make similar (but distinct) objects appear more similar than they actually are, thus promoting the perception of object stability^15^. Evidence for this mechanism is found in many domains. For example, perceived object orientation is systematically attracted toward the orientation of previous stimuli^15^. This attraction extends over a large temporal (10–15 seconds) and spatial range (20° of visual field), and shows a clear tuning in the orientation difference^15–18^. Similar serial dependence effects are found in the perception of faces^16, 19^, attractiveness^20–22^, ambiguous objects^23, 24^, numerosity perception^25^ and mapping numbers onto space^26^. Continuity fields may underlie some or all of these effects, and although the resulting perceptions may be less accurate in virtue of the bias introduced by serial dependence, they are more stable. In addition to perceptual stability, continuity fields reduce the need to constantly re-represent similar content over time.

Serial dependence occurs between single, isolated objects or features, but it is unknown whether serial dependence can occur between the global representations our visual system typically encounters in noisy, complex scenes. Here, we found evidence for serial dependence in ensemble representations, suggesting that it is a viable mechanism for promoting perceived stability of global, ensemble statistical information in scenes. This kind of serial dependence occurs at a level where the local signals are already integrated, and it is independent of the number of objects presented.

## Results

### Experiment 1: Serial dependence in ensemble representations

**Figure 1:**
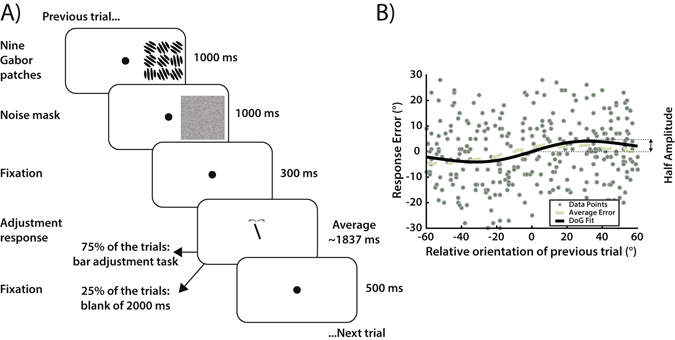
Experiment 1, trial sequence and data analysis. (**A**) Trial sequence for the method of adjustment task in Experiment 1. On each trial, a 3 × 3 grid of nine Gabors was presented for 1000 ms, followed by a 1000 ms noise mask of black and white pixels (to reduce afterimages) and a 300 ms fixation dot. Subjects were then asked to report the perceived average orientation of the Gabor array by adjusting the orientation of a response bar (75% of the trials) or to keep fixating the dot for an additional 2000 ms (25% of the trials). After a 500 ms delay, the next trial started. (**B**) Example data from Subject 2, with each data point showing performance on one trial. The x-axis represents the difference between the previous mean orientation and the current mean orientation. The y-axis represents the error in the adjustment task (difference between bar orientation and mean orientation on current trial). The average error (dashed line) shows more negative response errors for a negative relative orientation and more positive errors for a positive relative orientation. In order to quantify the magnitude of serial dependence, we fit a derivative-of-Gaussian (DoG) to the data (black line) measuring the half-amplitude peak for each observer.

To test for serial dependence in ensemble representations, we presented 3 × 3 arrays of nine randomly tilted Gabors in the peripheral visual field (Fig. 1). The standard deviation of the nine Gabors’ orientations was 10° on each trial, and the mean was selected randomly from a uniform distribution. We then measured the perceived ensemble orientation of each Gabor array using a method of adjustment task. Subjects were instructed to maintain fixation on a white dot during the experiment. On each trial, the Gabor array was presented for 1 s, after which a noise mask was presented for 1 s at the same location (Fig. 1A). After a 300-ms delay, in 75% of the trials a bar appeared at the fixation point location, and subjects were asked to adjust its orientation to match the perceived ensemble orientation of the Gabor array. In the remaining 25% of the trials, no response bar appeared and subjects were asked to keep fixating on the dot without responding (2 s delay).

**Figure 2:**
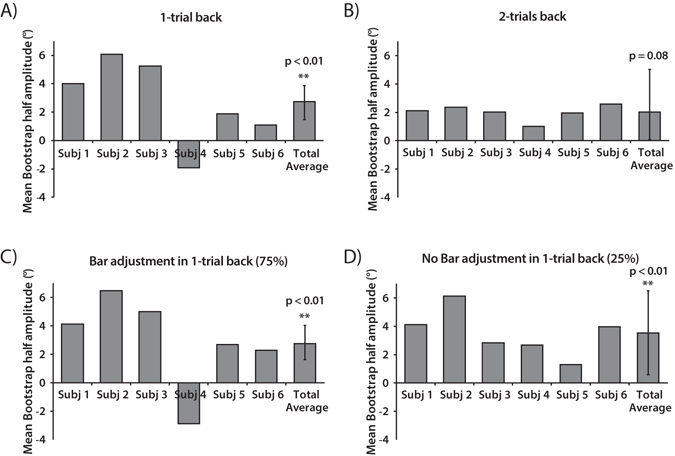
Mean bootstrapped half-amplitudes in Experiment 1. For each observer we obtained a mean bootstrapped half-amplitude by resampling the data with replacement 5000 times. Error bars on the Total Average are bootstrapped 95% confidence intervals, and the p value is based on the group null distribution. (**A**) Serial dependence half-amplitudes 1-trial back. (**B**) Serial dependence half-amplitudes 2-trials back. (**C**) Serial dependence half-amplitudes with bar adjustment 1-trial back. (**D**) Serial dependence half-amplitudes without bar adjustment 1-trial back.

Response error was calculated as the difference between the response bar orientation and the mean orientation of the Gabor array. We compared each subject’s error on the current trial to the difference in mean orientation between the current and previous trial (Fig. 1B). For each subject, we fit a simplified derivative-of-Gaussian (DoG). We then bootstrapped each subject’s data with 5000 iterations and reported the mean bootstrapped half-amplitude as a metric of the sequential dependence (Fig. 2).

All subjects except for one displayed a positive DoG half-amplitude, indicating that perceived ensemble orientation on a given trial was significantly pulled in the direction of ensemble orientation presented in the previous trial (p < 0.01, n = 6, group permuted null, Fig. 2A). Even when comparing subjects’ errors with the difference in orientation two trials back, all subjects showed trending positive DoG half-amplitudes (p = 0.08, one tailed, n = 6, group permuted null, Fig. 2B), meaning that even Gabor arrays presented two trials back biased perceived ensemble orientation on a given trial. Average response time across subjects was 1837 ± 0.57 ms. The perceived ensemble orientation of the Gabor array was therefore strongly attracted toward ensemble orientations seen more than 5 seconds (1 trial-back, Fig. 2A) or 10 seconds ago (2 trials-back, Fig. 2B).

However, serial dependence may be induced by the bar in the previous trials, which was adjusted to match the perceived ensemble orientation of the Gabor array. In order to control for this confound, we analyzed the trials where previous trials had no response bar (25% of the trials) and the current trials had a response bar (75% of the trials). The condition without bar adjustment still showed positive DoG half-amplitudes (p < 0.01, n = 6, group permuted null; Fig. 2C,D), meaning that serial dependence occurs between the average orientation of the Gabor arrays and the orientation of the response bar is not involved.

**Figure 3:**
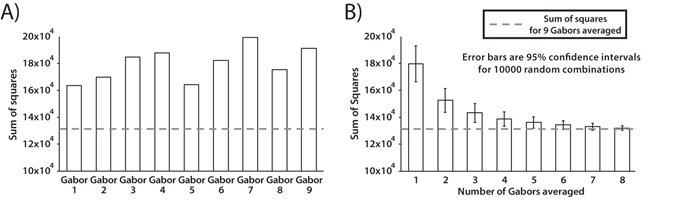
Sum of the squares analysis for Subject 2 in Experiment 1. Dashed lines indicate sum of the squares of the derivative-of-Gaussian fit for the average of nine Gabors (the empirically measured SSE for a plot like that in Fig. 1B). White bars indicate sum of the squares of the derivative-of-Gaussian fit for (**A**) single Gabors or (**B**) averages of subsets of Gabors. (**A**) The sum of the squares for the mean orientation is lower than any single Gabor, meaning that the observers did not match the bar orientation using a single Gabor. (**B**) The empirically measured sum of the squares for the mean is similar to the sum of the squares expected from a sample of five or more Gabor patches that have been averaged. This indicates that subjects averaged at least five Gabors. The mean of nine Gabors predicted responses significantly better than 5 or fewer Gabor patches for one subject, 4 or fewer for three subjects and 3 or fewer for two subjects.

In order to investigate whether serial dependence occurs between ensemble average representations (and not between single Gabors), we analyzed the sum of squared error (SSE) of the derivative-of-Gaussian fit (Fig. 1B). For each observer, we first determined the individual SSE for the DoG half-amplitudes plotted in Fig. 2A. We then determined the SSE for the individual Gabors. To do this, for each trial we plotted on the x-axis the difference between the previous orientation of a particular Gabor in the array and its current orientation. On the y-axis, we plotted the adjustment error relative to the particular Gabor. For each observer, we fit nine DoGs, one for each Gabor orientation in the stimulus array, and determined the SSE. We then compared the SSE for the data plotted as a function of the mean orientation with the SSE for the data plotted as a function of each single Gabor (Fig. 3A, dashed line vs. white bars).

If observers adjusted the bar orientation to match the orientation of a single Gabor (instead of the mean), the SSE for the data plotted relative to the single Gabor should be the lowest. However, the results show that the SSE for the data plotted relative to the mean orientation (Fig. 3A, dashed line) was lower than the SSE calculated from any of the single Gabors. These results indicate that serial dependence was most closely following the mean of the nine orientations. Hence, observers did not adjust the bar to match the orientation of a single Gabor.

Next, we conducted a simulation to examine how many possible Gabors contributed to serial dependence in the Gabor array. For each observer, we determined the SSE, as above, based on averaging different numbers of Gabors on each trial (1–8 total Gabors averaged). The relative orientation of the previous trial (x-axis), and response error (y-axis), were plotted for each possible Gabor average (e.g., an average of two, three, four, or more Gabors) instead of the mean of the entire array. That is, we re-plotted Fig. 2B with the average of n Gabors, where n could vary between 1 and 8; we determined the SSE of the DoG fit for each average. As different averages of n Gabors are possible within an array of 9 Gabors, we simulated 10000 possible combinations for each average. The results show that the SSE gradually decreased with an increasing number of averaged Gabors. For all observers, the SSE of the averaged Gabors equated the SSE of the mean of the array once the simulation reached an average of 4–6 Gabors (6 for one subject, 5 for three subjects and 4 for two subjects). Hence, the observers averaged groups of at least 4–6 Gabors per array. As this simulation does not assume any late stage or averaging noise, the estimated number of integrated Gabor patches is conservative.

### Experiment 2: Serial dependence occurs on the ensemble level

**Figure 4:**
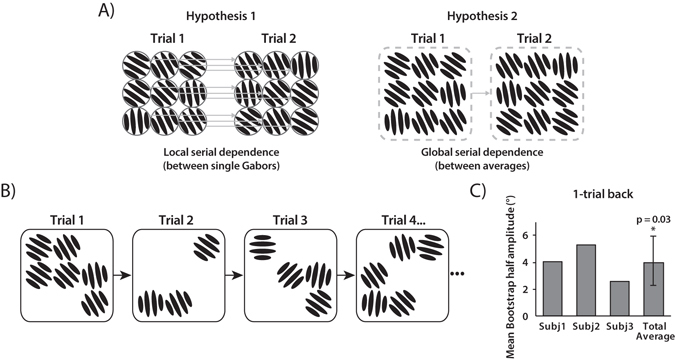
Experiment 2 stimuli and results. (**A**) Serial dependence may occur between single Gabors, following a retinotopic local correspondence (Hypothesis 1), or between orientation averages independent of retinotopic correspondence (Hypothesis 2). (**B**) From trial to trial, the Gabors were always presented in different numbers (from three to six) and spatial locations. (**C**) Mean bootstrapped half-amplitudes in Experiment 2 (1-trial back). Error bars are bootstrapped 95% confidence intervals, and the p-value is based on group null distribution.

In Experiment 2, we tested whether the perceptual pull across Gabor arrays occurred at the level of each individual Gabor (local serial dependence; Fig. 4A, left panel) or at the level of the ensemble percept of the Gabor array (global serial dependence; Fig. 4A, right panel).

In order to disentangle between these two hypotheses, we ran Experiment 1 with the following changes. First, on each trial, the number of Gabors presented in the 3 × 3 grid varied randomly from three to six. Second, the location of the Gabors changed from trial to trial so that no Gabor was presented at the same location in two consecutive trials (Fig. 4B). On each trial, observers were asked to adjust the bar orientation to match the perceived ensemble orientation of the three, four, five or six Gabors presented. If serial dependence occurs on a local level, the lack of correspondence between the single Gabors should disrupt any serial dependence effect on the mean orientation of the Gabor arrays. Conversely, if serial dependence occurs on a global level, serial dependence should still occur even without a correspondence between the single Gabor locations across trials. The results show that serial dependence occurs even when there is no correspondence on a local level (1-back trial: p = 0.03, n = 3, group permuted null, Fig. 4C; 2-back trial: p = 0.09, one tailed). Hence, serial dependence occurs between the global ensemble orientation of the Gabor arrays, at a level where local orientations are already merged into an ensemble.

### Experiment 3: Serial dependence between single and ensemble percepts

**Figure 5:**
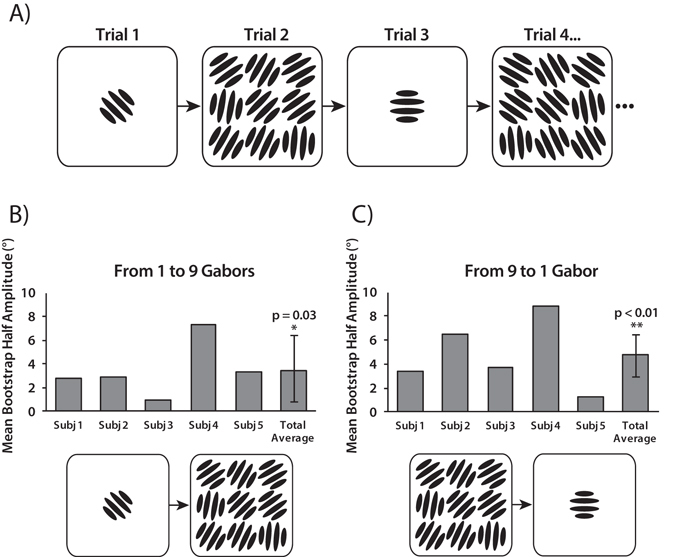
Experiment 3 stimuli and results. (**A**) A single Gabor alternated with an array of Gabors from trial to trial. When a single Gabor was presented, observers were asked to adjust the bar to match the single Gabor’s orientation. When nine Gabors were presented, observers were asked to adjust the bar to match the ensemble (average) orientation. (**B**–**C**) Mean bootstrapped half-amplitudes in Experiment 3 for serial dependence from a single Gabor orientation to an ensemble percept (**B**) and vice versa (**C**). Error bars are bootstrapped 95% confidence intervals, and p-value is based on group null distribution.

We have shown that serial dependence can occur in ensemble representations, providing a mechanism that could promote visual stability in complex environments. In Experiment 3, we test whether serial dependence can also occur between ensemble and individual object percepts. The procedure for Experiment 3 was identical to that of Experiment 1, except for the following changes. In 50% of the trials, subjects were presented with a single Gabor (at the center of 3 × 3 grid), and were asked to adjust the response bar to match its orientation. In the other 50% of the trials, subjects were presented with the 3 × 3 Gabor array, and were asked to adjust the bar to match the ensemble orientation. These two trial types alternated throughout each block (Fig. 5A).

We then analyzed the influence of the single presented Gabor on the percept of the Gabor array on the subsequent trial, and vice versa. The results show that serial dependence occurs in both conditions: single Gabor perception is biased towards the ensemble percept of the Gabor array on the 1-back trial (p = 0.03; n = 5 group permuted null; Fig. 5B), and the ensemble perception of the Gabor array is biased towards the single Gabor (p < 0.01; n = 5 group permuted null; Fig. 5C). Similar serial dependence was found 2-trials back (Gabor array: p = 0.01; Single Gabor: p = 0.03; n = 5 group permuted null).

We also investigated how many Gabors the observers averaged in the 3 × 3 Gabor array condition. For each observer, we first determined the standard deviation of the response error distribution relative to the average of nine Gabors (average of the mean). We then determined the standard deviation of the response error relative to different averages of Gabors (couples, triple, quadruplets and so on). As multiple combinations of averages are possible within an array of nine Gabors, we simulated 10000 possible combinations for each number of Gabors averaged.

Standard deviation of the response error gradually decreased with increasing number of Gabors averaged. Subjects responses were best predicted by the average of all the Gabors. To quantify the minimum number of Gabors integrated by each subject we compared the predictive power of the mean of randomly selected sets of n Gabors to the predictive power of all the nine Gabors. The mean of nine Gabors predicted responses significantly better than 2 or fewer for one subject, 4 or fewer for three subjects and 6 or fewer for one subject. Hence, observers averaged at least three Gabors in the 3 × 3 Gabor array condition. Our results confirm that serial dependence in complex contexts and can go from many-to-one and vice versa. Hence, serial dependence can occur between local and global properties of scenes.

## Discussion

Scenes appear to have stable identities and content from moment-to-moment despite changes in lighting, noise, discontinuities like eye blinks, and changes in gaze and head position. Our results reveal a possible mechanism that could promote this kind of apparent stability: a serial dependence in the perception of summary statistical information. Summary statistical information, such as average orientation, is one way in which observers perceive the “gist” of scenes^1, 3^, and our results show the perception of summary statistical information is sequentially dependent. Ensemble content in a scene, therefore, can be perceptually pulled toward previously viewed, similar ensemble scene information.

Recent results showed that perception of individual features or objects such as orientation^15^, faces^16^ and ambiguous objects^23, 24^ is systematically attracted by stimuli presented up to 10–15 seconds in the past. It was proposed that similar features and objects that are sequentially viewed are merged into a similar percept through a continuity field, a spatio-temporal integration mechanism with the aim of promoting object continuity^15, 16^. On a neural level, this has the advantage of reducing potential neural computations across time for each perceived object by recycling previously perceived features and objects. On a perceptual level, the continuity field helps us perceive a continuous and stable representation of the world despite noise and change. However, previous work only showed that serial dependence occurred between single, isolated objects. It remained unknown whether serial dependence could promote visual stability in the cluttered environment we experience everyday. Our results reveal serial dependence in ensemble representations, suggesting a mechanism through which continuity fields can maintain consistency of scene appearance on a moment to moment basis.

Our results show that ensemble orientation perception was pulled by ensemble percepts encountered five or ten seconds previously (Experiment 1). Importantly, this serial dependence did not depend on the subject’s response in the previous trial. In order to determine whether serial dependence occurs at the ensemble level, we manipulated the number and local spatial correspondence of the single Gabors in the Gabor array (Experiment 2). We found that serial dependence occurred even in the absence of local correspondence, meaning that serial dependence occurred at a level where orientations were already merged into global representations. Finally, we showed that single object properties influenced the subsequent perception of an ensemble, and an ensemble influenced the subsequent perception of a single object (Experiment 3). This provides evidence that serial dependence can operate across local and global levels of representation in a scene, such as between single elements and global ensembles.

Serial dependence in ensemble representations may also positively contribute to the phenomenon of change blindness in scenes. In a complex scene our visual system tends to represent visual information in terms of ensemble representations^1^, which often means losing or lacking high fidelity representations of individual objects in favor of representing the summary statistical information in the scene as a whole. For example, we perceive a lawn of grass, a shelf of books, or a crowd of runners. The individuals that make up each crowd can be lost or forgotten^27–30^ or crowded from awareness^7, 31^ but the visual system easily tracks the ensemble. If ensemble-level information is the critical level of perceptual awareness^32^ then losing the individual objects (even ones that change) may not be so costly. If this blade of grass or that book changes its features, it will not likely change the summary statistical information in the crowd as a whole. As the ensemble representation of a scene remains largely invariant to local modifications, observers may fail to notice changes in single objects simply because the visual system represents crowds and scenes at the level of the ensemble, not the particular. Further, if the ensemble-level information is somehow changed, the apparent magnitude of that change would be reduced by the serial dependence we report here. Sequential scenes would therefore appear more similar than they actually are, even if individual objects were to change. Hence, serial dependence in ensemble representations may contribute to the phenomenon of change blindness, and also play a crucial role in determining the appearance of consistency in sequentially viewed scenes.

Within a scene, our visual system has to stabilize its percept on multiple levels: from single features, to ensemble representations within the same feature, to a more global scene level. While we propose serial dependence in ensemble representations as a mechanism that promotes scene stability, we also acknowledge that our stimuli (3 × 3 arrays of Gabors) do not necessarily generalize to the real world scenes we experience everyday. However, our results are a first important step towards showing the impact of serial dependence on real scenes, from single features^15, 16, 19–22^ to ensemble representations. Future research will investigate whether serial dependence can occur on an even more global, naturalistic scene level.

Our finding of serial dependence in ensemble representations is also generally relevant for many experiments on visual perception. Often, visual stimuli are presented in rapid sequence under the assumption that each stimulus is perceived independently of other stimuli. Here, we showed that the perception of a series of complex stimuli can be biased by both local and global properties of stimuli presented in the previous 5–10 seconds. This result should be taken into consideration anytime perceptual experiments involve presenting sequences of complex stimuli.

## Conclusion

Our results show evidence for serial dependence between ensemble representations at a global level of integration. By merging similar ensemble percepts over time, the continuity field avoids reprocessing all of the local features of complex crowds and can promote visual stability in complex environments.

## General Methods

All experimental procedures were approved by and conducted in accordance with the guidelines and regulations of the UC Berkeley Institutional Review Board. Participants were affiliates of UC Berkeley and provided informed consent in accordance with the IRB guidelines of the University of California at Berkeley.

All participants had normal or corrected-to-normal vision, and all except one were naïve to the purpose of the experiment. Six subjects (4 females; age = 24–31 years) participated in Experiment 1. Three subjects (2 females; age = 24–31 years) participated in Experiment 2. Five subjects (4 females; age = 20–31 years) participated in Experiment 3.

Stimuli were generated on a Macintosh computer running Matlab PsychToolbox^33, 34^ and presented on a gamma-corrected CRT Sony Multiscan G500 monitor. The refresh rate of the display was 100 Hz and the resolution 1024 × 768 pixels. Stimuli were viewed from a distance of 57 cm. Subjects used a keyboard for all responses (left-right arrow keys to adjust the bar, and space bar to confirm bar orientation and initiate the next trial).

### Experiment 1

We presented a 3 × 3 array of nine tilted Gabors at 16° of eccentricity on the right visual field. Eccentricity refers to the distance from the fixation dot to the center of the central Gabor. Inter-spacing between Gabors was 2.5°. On each trial, Gabor orientations were approximately uniformly distributed with standard deviation 10° and a mean that was randomly selected from all possible orientations. The Gabors (windowed sine wave gratings) had a peak contrast of 65% Michelson, a spatial frequency of 2.4 cycles per degree, and a 0.35° s.d. Gaussian contrast envelope. A 0.2° diameter white dot served as a fixation point and subjects were instructed to maintain fixation for the duration of each experiment while performing the task. Gabors were presented for 1 s, after which a noise mask of randomly shuffled black and white pixels was presented for 1 s at the same location. The noise mask was a 7.5°-by-7.5° square presented at the Gabor grid location. After a 300-ms delay, in 75% of the trials a response bar (width: 0.5°, length: 4°, color: dark-gray) appeared at the fixation point location, and subjects were asked to adjust its orientation to match the perceived ensemble orientation of the Gabor array using the left/right arrow keys. The starting orientation of the bar was randomized on each trial. Subjects were allowed to take as much time as necessary to respond and pressed the spacebar to confirm the chosen bar orientation. In the remaining 25% of the trials, no response bar appeared and subjects were asked to keep fixating the dot with no response for 2 additional seconds. After a 500 ms delay, the next trial started. Each block was composed by 160 trials, for a total of 7 blocks (i.e., 1120 trials).

### Experiment 2

The experiment was the same as Experiment 1, with the following changes. The number of Gabor presented on the 3 × 3 grid varied from trial to trial from three to six, and it was never the same in two consecutive trials. In addition, the Gabors were never presented at the same location in two consecutive trials. Each block was composed of 100 trials, for a total of 4 blocks (400 trials total).

### Experiment 3

The methods were the same as Experiment 1, with the following changes. We alternated two types of Gabor configurations. In Gabor configuration A, a single Gabor was presented at 16° of eccentricity, i.e. the location of the central Gabor in the 3 × 3 grid. Subjects were asked to adjust the bar to match the orientation of the Gabor. In Gabor configuration B, nine Gabors were presented. Subjects were asked to adjust the bar to match the average orientation of the 9 Gabors. The trials were alternated following an A-B-A-B sequence. Each block was composed of 100 trials, for a total of 8 blocks (800 trials total).

### Data Analysis

#### Serial dependence fit

In Experiments 1–2, we measured subjects’ errors on the adjustment task to determine whether a subject’s perception of each ensemble orientation was influenced by the previously seen ensemble orientation. Response errors were computed as the difference between the response bar orientation and the averaged Gabor orientation (current bar orientation – current mean; y-axis on Fig. 1B).

Trials were considered lapses and excluded if errors exceeded −/+ 2 standard deviations from mean (on average, less than 5% of data excluded). As a measure of performance on the bar adjustment task, we computed the standard deviation of the response error for each observer and averaged across subjects. Experiment 1: mean 15.16° s.e. 1.36°; Experiment 2: mean 13.68° s.e. 1.42°; Experiment 3: Single Gabor: mean 14.06° s.e. 1.32°; Gabor array: mean 16.51° s.e. 2.24°. Relative orientation of the previous trial was computed as the difference between the previous mean orientation and the current mean orientation (previous mean– current mean). See x-axis on Fig. 1B. In Experiment 3, we investigated whether a subject’s perception of an ensemble orientation was influenced by the previously seen single orientation (Fig. 5B) or whether a subject’s perception of a single orientation was influenced by the previously seen ensemble orientation (Fig. 5C). In Fig. 5B, response errors were computed as the difference between the bar orientation and the averaged Gabor orientation (current bar orientation – current mean). Relative orientation of the previous trial was computed as the difference between the previous Gabor orientation and the current mean orientation (previous Gabor orientation – current mean). In Fig. 5C, response errors were computed as the difference between the matched bar orientation and the single Gabor orientation (current bar orientation – current Gabor orientation). Relative orientation of the previous trial was computed as the difference between the previous mean orientation and the current Gabor orientation (previous mean – current Gabor orientation).

In all experiments, we fit a simplified Gaussian derivative (DoG) to each subject’s data using the following equation:

$${\rm{y}}={{\rm{abcxe}}}^{-{({\rm{bx}})}^{2}}$$

where parameter *y* is response error on each trial, *x* is the relative orientation of the previous trial, *a* is half the peak-to-trough amplitude of the derivative-of-Gaussian, *b* scales the width of the Gaussian derivative, and *c* is a constant $\sqrt{2}/{{\rm{e}}}^{-0.5}$, which scales the curve to make the a parameter equal to the peak amplitude (Fig. 1B). We fit the Gaussian derivative using constrained nonlinear minimization of the residual sum of squares. As a measure of serial dependence, we reported the peak-to-trough amplitude of the derivative-of-Gaussian (parameter a; Figs 2, 4C, 5B and C).

#### Mean bootstrapped half amplitude

For each subject’s data, we generated confidence intervals by calculating a bootstrapped distribution of the model-fitting parameter values by resampling the data with replacement 5000 times^35^. On each iteration, we fit a new DoG to obtain a bootstrapped half-amplitude and width for each subject. We used the half amplitude of the DoG, the a parameter in the above equation, to measure the degree to which subjects’ reports of orientation were pulled in the direction of n-back mean/single orientations. For example, if in Experiment 1 subjects’ perception of the mean orientation was repelled by the 1-back mean orientation (e.g., because of a negative aftereffect), or not influenced by the 1-back mean (because of independent, bias-free perception on each trial), then the half- amplitude of the DoG should be negative or close to zero, respectively.

#### Permutation analysis

In order to calculate significance, we also generated a null distribution of the half amplitude (a) values for each subject using a permutation analysis. We randomly shuffled each subject’s response errors relative to the difference between the current and previous trial and recalculated the DoG fit for each iteration of the shuffled data. We ran this procedure for 5000 iterations in order to generate a within-subject null distribution of half amplitude values. P-values were calculated by computing the proportion of half amplitudes in each subject’s null distribution that were greater than or equal to the observed half amplitude.

#### Sum of the squares analysis (Experiment 1)

In Experiment 1, we determined the sum of the squared error (SSE) for the Gaussian derivative fit of each observer using the mean orientation from each trial (SSE of the mean). All the trials, with and without bar adjustment task, were taken into consideration. We also determined the SSE for the Gaussian derivative fit taking only single Gabor orientations into account (SSE of the single Gabors). On the y-axis, response errors were computed as “current bar orientation – current Gabor n”. On the x-axis, relative orientation of the previous trial was computed as the difference between the previous Gabor n orientation and the current Gabor n orientation. The comparison between SSE of the mean and SSE of the single Gabors is shown in Fig. 3A. Gabor numbers correspond to center (1), center-left (2), center-right (3), center-down (4), center-up (5), up-left (6), down-left (7), up-right (8), and down-right (9).

In order to investigate how many Gabors the observers were averaging, we simulated, for each observer, the SSE for the Gaussian derivative fit for averages of different numbers of Gabors (couplets, triplets, quadruplets and so on). On the y-axis, response errors were computed as “current bar orientation – current average of n Gabors”. On the x-axis, relative orientation of the previous trial was computed as the difference between the previous average of n Gabors and the current average of n Gabors. As multiple averages are possible within a 3 × 3 Gabor array, we simulated 10000 possible combinations for each average of n Gabors. Results from Subject 2 are shown in Fig. 3B. Error bars indicate the confidence intervals.

#### Standard deviation analysis (Experiment 3)

In Experiment 3, we first determined, for each observer, the standard deviation of the response error for the average of nine Gabors. We then simulated the standard deviation of the response error for different averages of Gabors (2–8 Gabors). Response errors were computed as “current bar orientation – current average of n Gabors”. As multiple averages are possible within a 3 × 3 Gabor array, we simulated 10000 possible combinations for each average of n Gabors.
